# Foodborne Botulism Type F: A Rare Presentation of Neurologic Manifestation and Successful Management

**DOI:** 10.7759/cureus.62964

**Published:** 2024-06-23

**Authors:** Nikky Maharjan, Yashitha Chirumamilla, Bibek Karki, Mohammed Berrou, Philip J Mcdonald

**Affiliations:** 1 Internal Medicine, Hurley Medical Center, Flint, USA; 2 Internal Medicine, Mayo Clinic, Rochester, USA; 3 Pulmonary and Critical Care Medicine, McLaren Flint, Flint, USA

**Keywords:** clostridium botulinum, neurotoxin, descending paralysis, botulism, botulism anti-toxin

## Abstract

Botulism is a neuroparalytic syndrome resulting from the systemic effects of an exoneurotoxin produced by gram-positive, rod-shaped, spore-forming, obligate anaerobic bacterium *Clostridium botulinum*. Here, we present the case of a 40-year-old male, presenting with a sudden onset of abdominal pain associated with vomiting. He was admitted for conservative management once the CT of the abdomen and pelvis revealed partial small bowel obstruction with no signs of bowel perforation or ischemia. However, the next day, the patient had a cardiac arrest thought to be secondary to respiratory arrest. The return of spontaneous circulation was achieved after two cycles of cardiopulmonary resuscitation. The patient developed quadriplegia, areflexia, and bilateral ophthalmoplegia. He was empirically treated with pyridostigmine, intravenous immunoglobulin (IVIG), and botulinum antitoxin. Stool polymerase chain reaction (PCR) testing resulted positive for *C. botulinum* toxin type F. The patient ultimately recovered with botulinum antitoxin and a month of physical and speech therapy. Our case highlights that clinicians should consider botulism as a differential and emphasize the importance of early diagnosis for effective management and prognosis.

## Introduction

Botulism is a rare, potentially life-threatening disease caused by neurotoxins produced by *Clostridium *bacteria, particularly *Clostridium botulinum*. Among the eight recognized toxin types (A-H), A, B, and E are the most common to be associated with foodborne outbreaks; type F is far less frequently responsible [1,2]. The toxin blocks acetylcholine receptors at neuromuscular junctions, resulting in descending flaccid paralysis [3]. Type F botulism represents approximately 1% of botulism cases encountered in the United States [4].

## Case presentation

We present the case of a 40-year-old male with a past medical history of hypertension, prediabetes, dyslipidemia, gastritis, alcoholic liver disease, and alcoholic pancreatitis who presented to the ED with complaints of abdominal pain and vomiting. He described the abdominal pain to be intermittent and crampy in nature, initially in the upper quadrant but later diffuse. He further stated that this pain was different from his previous bouts of pancreatitis. He denied changes to his bowel habits. Upon presentation, he was found to be afebrile, hypertensive with a blood pressure of 164/107 mmHg, tachycardic with a heart rate of 125, and saturating well on room air. Physical examination was significant for mild diffuse lower quadrant abdominal tenderness. The initial laboratory evaluation was unremarkable. A CT of the abdomen and pelvis was suggestive of partial small bowel obstruction with no signs of bowel perforation or ischemia. Abdominal decompression was performed via a nasogastric tube, removing 400-500 mL of fluid. The next day, the patient had new complaints of dysphagia and a change in voice. With suspicion of a retropharyngeal abscess, a CT of the head and neck was planned, and, during the transportation, he was noted to have difficulty breathing leading to fatigue. His lips became cyanotic and his oxygen saturation dropped to the 70s on room air. He soon lost pulse, and chest compressions were initiated. His initial rhythm was pulseless electrical activity. Return of spontaneous circulation (ROSC) was achieved after several cycles of cardiopulmonary resuscitation (CPR) that lasted six minutes, and the patient was intubated, placed on a mechanical ventilator, and transferred to the ICU.

**Figure 1:**
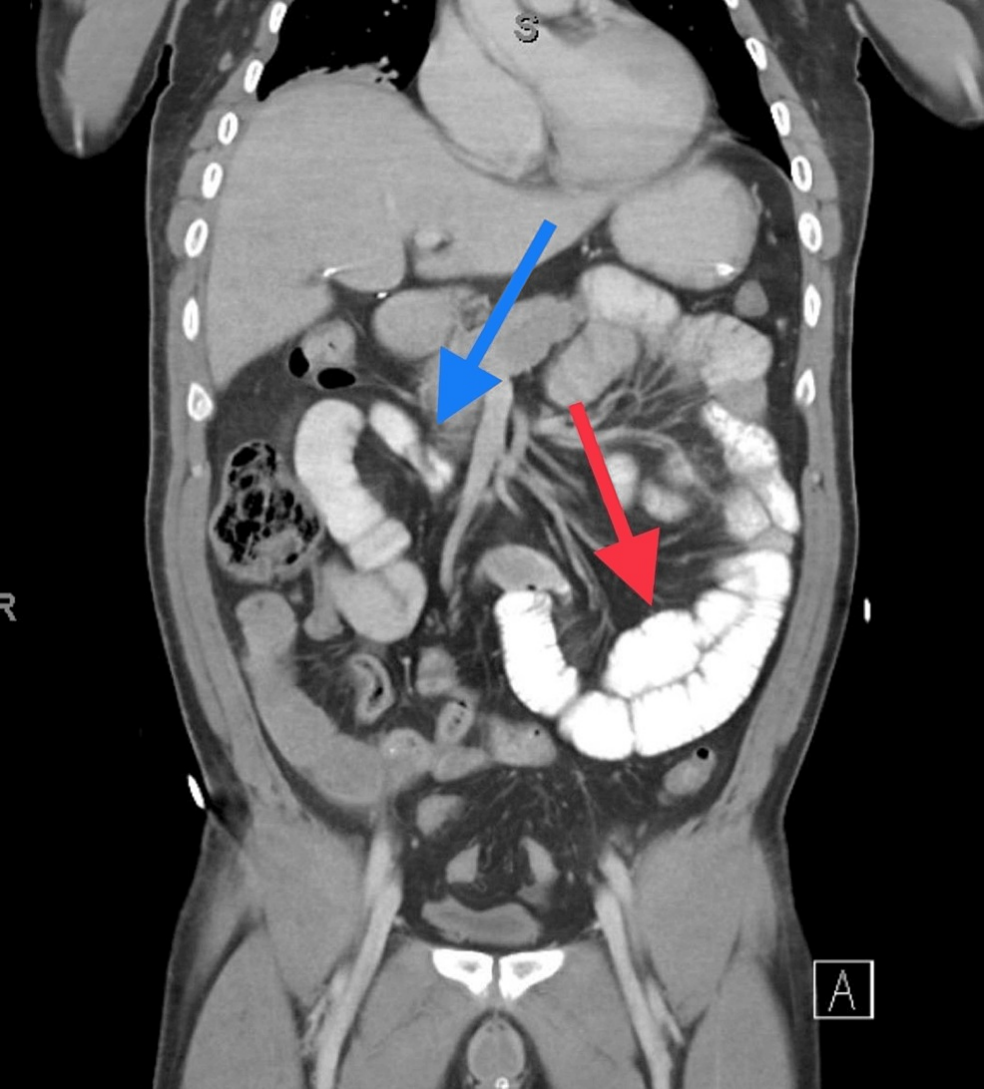
A CT scan abdomen pelvis showing small bowel obstruction with contrast within the dilated loops of the small bowel (red arrow) but no contrast noted within the distal decompressed small bowel loops (blue arrow).

Upon arrival to the ICU, he was hypothermic but alert and oriented. A post-cardiac arrest laboratory evaluation revealed a new onset hypokalemia with a potassium of 2.7 mEq/L (reference range: 3.4-5.3 mEq/L) and respiratory acidosis with a pH of 7.19 (reference range: 7.35-7.45) and a partial pressure of carbon dioxide (pCO^2^) of 65 mmHg (reference range: 32-48 mmHg). Subsequently, he developed progressive quadriplegia, areflexia, bifacial ptosis, and bilateral ophthalmoplegia with fixed dilated pupils. Neurology was consulted for further recommendations. They suspected an acute neuromuscular disorder in the setting of his sudden onset physical examination findings and acute respiratory failure. The differential diagnosis included botulism, Miller-Fisher syndrome, neurological manifestation of Lyme disease, West Nile virus infection, lead or other heavy metal poisoning, and tick paralysis. Although some of the diagnoses were less likely, a broad workup was done to assertively rule them out. While awaiting the results, the patient was empirically treated with pyridostigmine, intravenous immunoglobulin (IVIG), and botulinum antitoxin. Blood cultures showed no growth for five days. A lumbar puncture was done, and the CSF analysis revealed glucose to be 53 mg/dL (reference range: 30-70 mg/dL) and protein 71 mg/dL (reference range: 15-45 mg/dL). A CSF culture revealed no organism growth and a negative Lyme antibody. Acetylcholine receptor antibody was found to be negative. The blood test for Anti-GQ1b antibodies resulted negatively. The heavy metal screen was negative for the presence of lead, arsenic, and mercury. Ultimately, the stool polymerase chain reaction (PCR) testing detected the gene for *C. botulinum* toxin type F (Table 1). On further inquiry, he reported a history of frequent consumption of canned beans, assumed to be the source of his infection. Despite gradual neurological improvement, a successful extubation was not achieved, necessitating a tracheostomy. After a month of extensive physical and speech therapy, the patient underwent tracheostomy removal and recovered completely.

**Table 1:** Patient’s diagnostic lab workup demonstrating the stool polymerase chain reaction positive for Clostridium botulinum type F and all other tests negative for Miller Fisher syndrome, Myasthenia Gravis, West Nile virus, and heavy metal screen.

Diagnostic evaluations	Results
Clostridium botulinum toxin type F	Positive
GQ1b Antibody IgG	Negative
Acetylcholine receptor antibody	Negative
West Nile virus antibody	Negative
Heavy metal screen (lead, arsenic, mercury)	Negative

## Discussion

The epidemiology and clinical presentation of type F botulism have been studied the least compared to other neurotoxin types that are well known to cause the disease. A study was conducted in 2005 to further review reported cases of toxin type F. There were 1,269 cases of botulism among adults and infants that were reported to the Center for Disease Control and Prevention (CDC) between 1981 and 2002, none of which were part of an outbreak. Among those cases, only 13 (1%) were adult type F, and a toxigenic *Clostridium baratii* was identified in nine (69%) cases [5]. There has not been any data for *C. botulinum*-producing toxin type F as a causative agent. However, from the data reviewed by the CDC, it can be concluded that adult type F botulism is rare, and *C. botulinum* is even rare as a causative agent.

In a study published in 2016, they reviewed all the confirmed cases of botulism in the USA between 1979 and 2009 and found that toxin type F had higher mortality (13.8%) than type A, B, or E (range: 1.4%-4.1%). The clinical presentation of type F botulism was also noted to be more sudden in onset with quicker progression to respiratory failure and paralysis, as demonstrated in our patient. Type F botulism is also speculated to be secondary to intestinal colonization in adults with a prior history of gastrointestinal disease or recent antimicrobial use; hence, the higher mortality rate might be reflective of the worse overall health of those affected [6].

The only specific therapy for botulism is toxin-type specific botulinum antitoxin, which is an equine-derived antibody. The antibodies bind and neutralize the free botulinum toxin in the bloodstream forming the antitoxin-toxin complex, which is then cleared from the circulation. The antitoxin is effective if administered early in the course of illness, ideally within 24 hours or at least within 48 hours of symptom onset. However, patients can survive, even without antitoxin, if they receive supportive care including mechanical ventilation, which is reflected by a decrease in the mortality rate to <5% with the development of mechanical ventilation compared to the case fatality ratio of 70% in the first half of the 20th century [7].

Although a definitive diagnosis of botulism had not been made, the antitoxin was administered to our patient within 24 hours of the onset of his symptoms and resulted in a beneficial outcome. The adverse effects of the antitoxin administration that have been documented by the Food and Drug Administration (FDA) are infusion reactions, type I hypersensitivity, and serum sickness syndrome. Being a time-sensitive treatment, an interprofessional approach is recommended when considering and acknowledging the risks and benefits [8].

This case highlights the presentation and successful management of botulism caused by neurotoxin type F, a rare, neuroparalytic disease with significant mortality rates. A multidisciplinary approach with a broad differential, followed by appropriate diagnostic workup and prompt administration of botulism antitoxin was crucial in ensuring his ultimate recovery.

## Conclusions

Clinicians should consider botulism as part of the differential diagnosis when encountering patients presenting with neuroparalytic symptoms. The literature demonstrates that type F botulism has never occurred as part of outbreaks so even in the absence of history strongly suggesting botulism, it still should remain a differential. A retrospective inquiry allowed for possible source identification in our patient, but it remains ambiguous. Early suspicion is necessary for effective management and prognosis. In the case of our patient, the antitoxin was administered despite obtaining confirmation of botulism, and, ultimately, it appears to have been the correct clinical judgment because otherwise there would have been a significant delay in antitoxin administration, and the patient may not have attained a successful recovery.
